# Molecular Identification and Drug Sensitivity Test of *Acinetobacter lwoffii* from Cynomolgus Monkey with Peritonitis

**DOI:** 10.3390/vetsci13020170

**Published:** 2026-02-09

**Authors:** Heling Li, Ziyao Qian, Lan Luo, Hong Wang

**Affiliations:** 1 State Key Laboratory of Primate Biomedical Research, Institute of Primate Translational Medicine, Kunming University of Science and Technology, Kunming 650500, China; lihl@lpbr.cn (H.L.); qziyao_7yr@163.com (Z.Q.); rolland0606@163.com (L.L.); 2 Yunnan Key Laboratory of Primate Biomedical Research, Kunming 650500, China

**Keywords:** cynomolgus monkey, *A. lwoffii*, isolation and identification, drug sensitivity test

## Abstract

A case of peritonitis in a female cynomolgus monkey (*Macaca fascicularis*) caused by suspected bacterial infection was found. In order to identify the pathogenic bacteria, pathological tissues from the peritoneum of the animal were collected, and a bacterial strain designated MF080196 was isolated. Morphological observations were conducted, followed by biochemical testing and sequencing of its 16S rRNA gene. Additionally, pathogenicity test and drug sensitivity analyses were performed. The findings indicated that colonies grown on blood agar medium appeared white, circular, and raised; the bacteria were identified as Gram-negative; biochemical tests aligned with the characteristics of Acinetobacter lwoffii (*A. lwoffii*). PCR amplification was performed using a 16S rRNA sequence measuring 1443 bp; there was a remarkable similarity of 99.8% with previously reported sequences of *A. lwoffii* from various sources. The isolated strain MF080196 is capable of mortality in mice; furthermore, drug susceptibility testing revealed that the isolated strain exhibited high sensitivity to 23 antibiotics, including ceftriaxone, gentamicin, and levofloxacin. The pathogen isolated from the peritoneal tissue of the dead cynomolgus monkey was identified as *A. lwoffii*, which demonstrated sensitivity to most antibiotics tested in this study. This research provides significant insights for preventing and treating bacterial diseases in non-human primates.

## Simple Summary

A bacterial strain was isolated from the diseased peritoneal tissue of a dead cynomolgus monkey. It was identified as *Acinetobacter lwoffii* (*A. lwoffii*) by bacterial isolation and culture, morphological observation, physiological and biochemical examination and molecular biological methods. To our knowledge, this is the first report of the isolation of *A. lwoffii* from a peritonitis cynomolgus monkey. The results provide a reference for the prevention and treatment of bacterial peritonitis in experimental monkeys.

## 1. Introduction

Peritonitis is an inflammatory response of the peritoneum caused by bacterial infection, chemical irritation, or physical injury. It is also a prevalent disease among monkeys. Simon et al. [1] reported a case of a death cynomolgus monkey due to severe infection with peritonitis after dystocia; Takos and Thomas [2] isolated *Escherichia coli* and *Clostridium perfringens* from the exudate of peritonitis in marmoset monkeys; Clemmons et al. [3] documented a case of fibrinous purulent peritonitis in a rhesus monkey, which was accompanied by liver abscess and intestinal serositis; Hahn et al. [4] also reported a case of peritonitis in cynomolgus monkey caused by intestinal perforation. The pathogenic microorganisms involved in peritonitis are diverse, with common pathogens including *Escherichia coli*, *Klebsiella pneumoniae*, and *Staphylococcus aureus* [5,6]. Non-human primates are essential experimental animals for exploring human development, disease mechanism and the development of treatment technology [7]. Once animal peritonitis occurs, it can cause a series of clinical symptoms and even lead to death, which seriously threatens animal health.

The genus *Acinetobacter*, proposed by Bouvet and Grimont in 1986, includes seven named and nine unnamed genomospecies [8]. The genus *Acinetobacter* is not demanding on nutrition and is able to utilize a variety of different substances as a sole source of energy. As a result, these organisms are generally widely distributed and commonly found in soil, water, dry environments, as well as in hospitals. The organism can be found in a wide range of foods, including chicken meat, other meats and dairy products [9]. *A. lwoffii* is classified as a non-fermenting Gram-negative aerobic bacillus within the genus *Acinetobacter* [9], recognized as an emerging pathogen affecting both humans and animals, which can cause pneumonia, meningitis, urinary tract infection, septicemia, gastroenteritis and wound infection [10,11,12]. Furthermore, *A. lwoffii* has been widely identified across different animal species, including fish [13,14], chickens [15], birds [16], dogs [17], and alpacas [18]. However, there have been no prior reports documenting cases of cynomolgus monkeys infected with *A. lwoffii* leading to peritonitis until now. In this study, a strain of *A. lwoffii* was isolated from the abdominal cavity of a cynomolgus monkey with peritonitis, and the biochemical test, 16S rRNA gene genetic characteristics, pathogenicity test and drug sensitivity test of the strain were performed. This study investigates the isolation, identification, virulence, and antimicrobial susceptibility of Acinetobacter lwoffii isolated from a peritonitis case in a cynomolgus monkey to guide the prevention and treatment of bacterial peritonitis in macaques.

## 2. Materials and Methods

### 2.1. Samples

In this study, the female cynomolgus monkey was sourced from Kunming University of Science and Technology [SYXK(Yunnan) K2022-0001]. All procedures were approved by the Institutional Animal Care and Use Committee (IACUC) of Kunming University of Science and Technology (approval code: KUST202404003). The clinical symptoms observed in the animal included thin and scanty feces, reduced appetite, depression, and lethargy. A complete blood count, serum biochemistry, electrolyte analysis, and microscopic examination of the feces were performed when the animal presented with clinical symptoms. Venous blood samples were collected for complete blood count, serum biochemistry, and electrolyte analysis. The complete blood count indicated a total white blood cell count of 17.70 × 10^9^/L (reference range: 6.0–14.0 × 10^9^/L), an absolute neutrophil count of 11.04 × 10^9^/L (reference range: 4–10 × 10^9^/L), and a neutrophil percentage of 84.3% (reference range: 40–70%). Biochemical analyses showed a serum albumin level of 14 g/L (reference range: 28–44 g/L) and c-reactive protein (CRP) value of 15.9 mg/L (reference range: ≤9 mg/L), while other parameters remained within normal reference value. Blood test results revealed leukocytosis, neutrophilia, elevated CRP, and hypoalbuminemia, suggesting that this animal may have a continuous bacterial infection. Microscopic examination of the feces revealed a minor *Trichomonas* infection. The animal received treatment with oral metronidazole tablets at a dosage of 20 mg/kg/day and an intramuscular injection of cefazolin sodium at 50 mg/kg. Unfortunately, after two days of treatment, the animal’s condition deteriorated rapidly on the third day, and she died after ineffective treatment. Only three days elapsed between the onset of clinical symptoms and death.

### 2.2. Necropsy Examination

**Figure 1:**
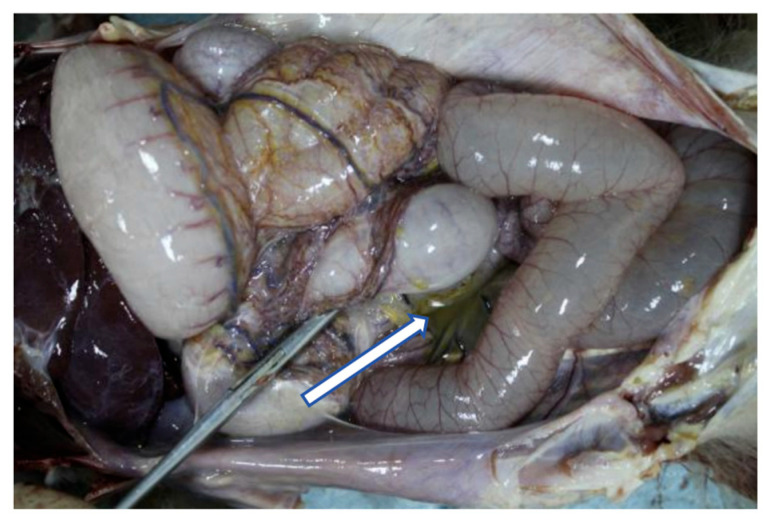
A general necropsy of the cynomolgus monkey with peritonitis in this study. Yellowish-green exudate in the abdominal cavity (indicated by the white arrow).

A necropsy was promptly conducted on the deceased animal, revealing yellowish-green exudate in the abdominal cavity (indicated by the white arrow) as well as yellowish sticky substances adhering to both mesentery and omentum (Figure 1). The peritoneal tissue and exudate samples were collected during autopsy.

### 2.3. Main Reagents and Instruments

Disposable sterile sampling swabs, Columbia blood agar medium, and Mueller–Hinton agar medium were purchased from Qingdao Hope Biological Co., Ltd., (Qingdao, China); antibiotic susceptibility disks were obtained from Changde Bkmam Biotechnology Co., Ltd., (Changde, China); 2× Taq PCR Master Mix was sourced from Tiangen Biotech Co., Ltd., (Beijing, China); a Gel Extraction Kit was acquired from Beijing Tsingke Biotech Co., Ltd., (Beijing, China); the Gram staining reagent kit was purchased from Beijing Solarbio Science & Technology Co., Ltd., (Beijing, China); DNA Marker 2K was supplied by Beijing TransGen Biotech Co., Ltd., (Beijing, China); microbial biochemical identification tubes were obtained from Hangzhou Microbial Reagent Co., Ltd., (Hangzhou, China). The PCR instrument utilized in this study was manufactured by Eppendorf Co., Ltd., (Hamburg, Germany); the DNA electrophoresis equipment came from Shanghai Tanon Science & Technology Co., Ltd., (Shanghai, China); and the constant temperature incubator was produced by Shanghai Yiheng Scientific Instrument Co., Ltd., (Shanghai, China). An ordinary optical microscope used for observations originated from Nikon Corporation (Tokyo, Japan).

### 2.4. Bacterial Isolation and Morphological Observation

A sterile swab was employed to collect yellowish-green exudate samples from the peritoneal cavity. The sample was mixed with physiological saline before being inoculated onto Columbia blood agar medium using an inoculation loop. The plates were placed separately in a regular incubator and an anaerobic incubator, and incubated at 37 °C for 24 h to observe the growth of bacteria. A single colony isolated on the medium was picked and smeared evenly onto a glass slide with a drop of physiological saline. Following drying, Gram staining procedures were conducted, allowing for morphological observation under an optical microscope at a magnification of 1000.

### 2.5. Biochemical Identification

The isolated bacterial strain underwent dilution in 0.9% physiological saline to achieve a turbidity equivalent to 0.5 McFarland standard (approximately 10^8^ CFU/mL). Subsequently, 10 µL of the bacterial suspension was added to a micro-biochemical identification tube CYZ-15E for the Enterobacteriaceae kit (Binhe Microorganism Reagent, Hangzhou, China, catalog: 250708), following operational protocols as outlined in the biochemical identification instructions. Use an inoculating needle to stab-inoculate the bacteria cultured in fresh meat broth into semi-solid agar medium. Take another tube with no bacteria as a blank control, and incubate both at 37 °C for 18~48 h to observe their growth and motility. Place a fresh pure culture colony on a clean glass slide; add 1–2 drops of 3% H_2_O_2_ and observe immediately. Producing a large amount of bubbles within 10 s indicates a positive result, while no bubbles or very few bubbles indicate a negative result. Take a fresh pure culture colony and smear it on an oxidase test strip. Purple-red/dark blue color appearing within 10~30 s indicates a positive result; color development after more than 1 min is a false positive, and no color change indicates a negative result.

### 2.6. Amplification and Sequencing of Bacterial 16S rRNA

In accordance with reference [19], universal bacterial primers (27F: 5′-AGAGTTTGATCCTGGCTCAG-3′ and 1492R: 5′-CGGCTACCTTGTTACGACTT-3′) were utilized for amplifying the gene encoding the 16S rRNA sequence. The PCR mixture comprised 12.5 µL of 2× Taq PCR Master Mix—the upper and lower primers (10 µmol/L) were 1 µL each—and was supplemented with distilled water up to a total volume of 25 µL. A small quantity of bacteria collected from a single colony on Columbia blood agar plate was utilized as the PCR template.

The programmed conditions for PCR amplification included initial denaturation at 95 °C for 3 min, followed by 35 cycles consisting of denaturation at 95 °C for 30 s, annealing at 55 °C for 30 s, and extension at 72 °C for 1 min. A final extension step occurred at 72 °C for 5 min. The PCR products were identified by 1.5% agarose gel electrophoresis for 30 min at 130 V and recovered, and sequencing was carried out by Beijing Tsingke Biotechnology Co., Ltd., in Beijing, China.

### 2.7. Sequence Alignment and Phylogenetic Tree Construction

Obtained gene sequences underwent comparison against related sequences available within NCBI GenBank employing BLAST methodology, Version 2.17.0. Following this analysis, the ClustalW method in MegAlign software integrated into MEGA11 (version 7.0.26) enables sequence alignment and constructing phylogenetic trees aimed at elucidating genetic relationships alongside evolutionary trajectories among *A. lwoffii* strains derived from various sources.

### 2.8. Animal Pathogenicity Test

Isolated *A. Lwoffii* (MF080196) was cultured on nutrient agar slant media, then incubated under controlled conditions set at 37 °C for durations of 18–24 h. The resultant colonies were subsequently washed off using sterile physiological saline. All mice were purchased from Shanghai Nanfang Model Biotechnology Co., Ltd., (Shanghai, China). The mice were kept in a barrier environment that met the Chinese national standards. The environmental conditions were strictly controlled: the temperature was maintained at 22 ± 2 °C, the relative humidity was 40%~70%, and the light cycle was 12 h of alternating light/dark. The mice had free access to radiation-sterilized feed and sterile drinking water, and the bedding materials were autoclaving shavings and corn cobs. All operations were performed in a biosafety cabinet to maintain a sterile state. Six healthy mice aged 10 weeks were randomly allocated into two groups containing three individuals each. In the experimental group, 3 mice were intraperitoneally injected with 1 × 10^5^ CFU, 1 × 10^6^ CFU, and 1 × 10^7^ CFU bacterial suspensions, respectively, while the control group mice were administered the same volume of saline solution via intraperitoneal injection [20]. After injecting the bacteria, the disease symptoms and the number of dead mice were observed and recorded every 12 h. Mice were evaluated for euthanasia if they lost more than 20% of their initial body weight or were unable to eat and drink independently. The mice were under normal feeding management for 7 days. The dead mice were immediately autopsied, and the surviving mice were euthanized by intraperitoneal injection of sodium pentobarbital at 150 mg/kg on the 7th day to observe the histopathological changes in the mice and isolate and culture bacteria from the diseased tissues of dead mice.

### 2.9. Antibiotic Susceptibility Test

Twenty-four commonly employed antibiotic susceptibility disks, including ceftriaxone, amikacin, gentamicin, ampicillin, cefazolin, ceftazidime, levofloxacin, cotrimoxazole, ciprofloxacin, norfloxacin, erythromycin, rifampicin, amoxicillin, furazolidone, cefoperazone, cefadroxil, ofloxacin, imipenem, meropenem, and azithromycin, present within our laboratory were selected for testing. Bacterial suspensions were diluted to achieve concentrations measured at 1.0 × 10^8^ CFU/mL utilizing McFarland standards. Under aseptic conditions, 100 µL of the bacterial solution was evenly coated in Mueller–Hinton agar medium plates. The drug susceptibility disks to be tested were taken with sterile tweezers and attached to the surface of Mueller–Hinton agar medium. The disks were cultured at 37 °C for 24 h, and the inhibition zone diameter of each drug susceptibility disk was measured. Kirby–Bauer (K-B) method was used to determine the drug sensitivity of the isolated strains according to the antimicrobial susceptibility test standards [21].

## 3. Results

### 3.1. Morphological Characteristics of Bacteria

**Figure 2:**
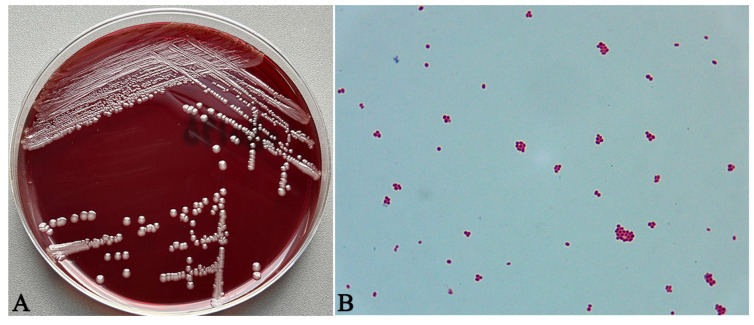
Morphological characteristics of isolated bacteria. (**A**) Morphological observations of the isolated strains on Columbia blood agar medium, characterized as white, round, raised, smooth, moist, with a diameter ranging from 1 to 2 mm. (**B**) Gram staining of the isolated strain (100×).

Only one type of colony was observed on the medium cultured in the regular incubator, while no colony growth was seen on the medium cultured under anaerobic conditions. The colonies were white, round, raised, smooth, moist, with a diameter ranging from 1 to 2 mm, and exhibiting stickiness (Figure 2A). Gram staining results revealed that the bacteria were Gram-negative, rod-shaped, and typically arranged in pairs or short chains (Figure 2B).

### 3.2. Biochemical Tests of Isolated Strains

The biochemical characteristics of the isolated strain MF080196 are summarized in Table 1. The results indicated that this strain was non-motile because the bacteria grew only along the stab line; it did not ferment sugars nor exhibit positive reactions for amino acid metabolism tests and VP and MR tests. Catalase reaction was positive and oxidase reaction was negative. Additionally, it did not produce hydrogen sulfide and could not utilize citrate as its sole carbon source. According to the identification criteria outlined in the “Handbook of Systematic Identification of Common Bacteria”, these biochemical reaction results align with those characteristic of *A. lwoffii* (Table 1).

### 3.3. Amplification and Sequence Analysis of 16S rRNA from Isolated Strains

#### 3.3.1. Results of 16S rRNA Amplification of Isolated Strains

**Figure 3:**
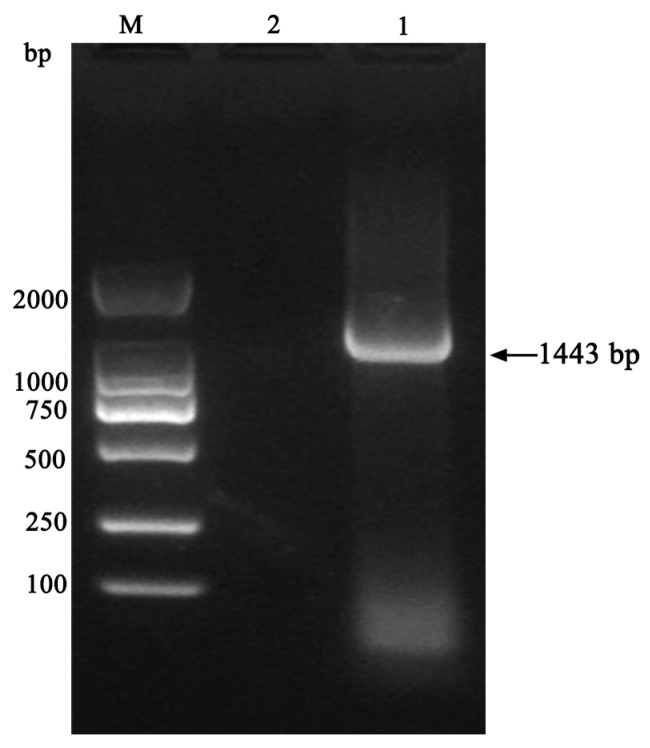
PCR amplification of the 16S rRNA gene of *A. lwoffii* from the cynomolgus monkey. (M: DL-2K Marker; 1: sample; 2: Negative control). The original PCR image can be seen in Figure S1.

PCR products were analyzed using 1.5% agarose gel electrophoresis, which yielded a target band within the range of 1000–2000 bp (Figure 3). The target gene band was subsequently purified and sequenced; the resulting sequence for the 16S rRNA was 1443 bp in length.

#### 3.3.2. Comparative Homology Analysis Between Isolated Strains and Various Sources of *A. lwoffii*

**Figure 4:**
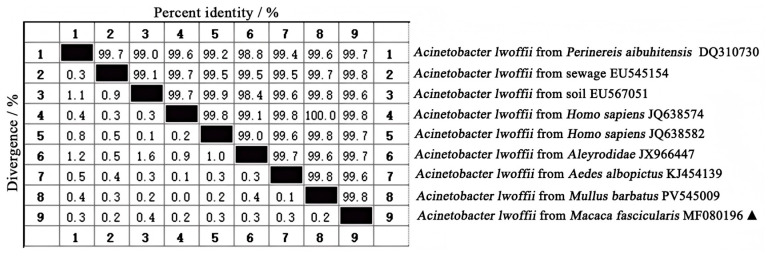
Homology comparison of isolated strains with *A. lwoffii* from different sources. “▲” is the isolated bacteria strain from the cynomolgus monkey in this study.

The obtained gene sequence underwent comparison analysis via MegAlign11 (version 7.0.26) software, revealing a similarity rate up to 99.8% with *A. lwoffii* strains, indicating that our isolated strain is indeed *A. lwoffii*, designated as MF080196 (PX928691). Furthermore, comparative sequencing against various sources available in NCBI GenBank demonstrated that similarities exceeded 99.6%, with the highest similarity was 99.8% when compared to sequences derived from sewage (EU545154), *homo sapiens* (JQ638574), and *mullus barbatus* (PV545009) (Figure 4).

#### 3.3.3. Phylogenetic Tree Analysis Based on Gene Sequences

**Figure 5:**
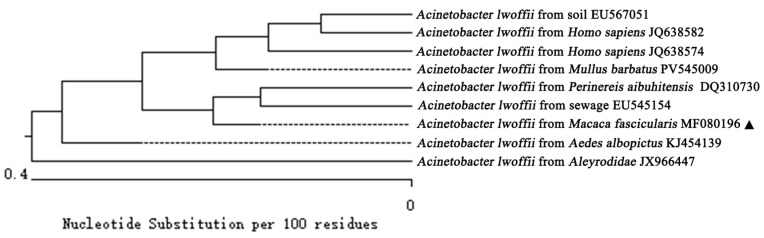
Phylogenetic tree of 16S rRNA sequences among different reported strains of *A. lwoffii,* including cynomolgus monkey (*Macaca fascicularis*) (MF080196). “▲” is the isolated bacteria strain from the cynomolgus monkey in this study.

Phylogenetic tree analysis results illustrated that nine strains identified as *A. lwoffii* from diverse species origins formed two principal genetic evolutionary branches (Figure 5). Notably, our isolated strain MF080196 (PX928691) clustered within the same branch alongside seven other strains sourced differently while forming a smaller sub-branch closely related to both *A. lwoffii* isolates from *Perinereis aibuhitensis* (DQ310730) and sewage samples (EU545154). Conversely, an isolate derived from *Aleyrodidae* (JX966447) occupied an independent genetic branch, demonstrating a more distant relationship relative to MF080196 (PX928691).

### 3.4. Results of Animal Pathogenicity Tests

Twenty-four hours after the injection of the bacterial liquid, the mice in the experimental group began to show symptoms such as listlessness, slow activity, loss of appetite, and diarrhea, and all died within 48 h. Culture characteristics and morphological features consistent with strain MF080196 were isolated from the dead mice. Dissection of mice infected with *A. lwoffii* showed congested and edematous peritoneum, with pale yellow turbid exudate in the abdominal cavity; the liver and spleen were enlarged and dark red in color. However, the mice in the control group were healthy within 7 days, and no gross lesions were found in the internal organs during post-mortem examination.

### 3.5. Antimicrobial Susceptibility Test Results of the Isolated Strain

The susceptibility test results indicate that this isolated strain, MF080196, was sensitive to 19 antibiotics, including ceftriaxone, amikacin, gentamicin, ampicillin; moderate sensitivity was observed towards cefazolin (Table 2).

## 4. Discussion

*A. lwoffii*, recognized as a significant opportunistic pathogen [9], exhibits diverse pathogenic mechanisms. These include the continuous aggregation and activation of neutrophils and macrophages through the direct secretion of cytokines such as IL-8; alternatively, *A. lwoffii* can directly interact with epithelial cells to stimulate cytokine release, thereby promoting epithelial cell proliferation [7]. Despite its high infection rates in humans and animals, reports of *A. lwoffii* infections in non-human primates are scarce [22]. In this study, a bacterial strain was isolated from the abdominal cavity of the cynomolgus monkey diagnosed with peritonitis. The strain was identified as *A. lwoffii* via morphological assessment, biochemical testing, and 16S rRNA gene analysis; furthermore, pathogenicity and drug sensitivity tests were conducted on the isolated strain to provide critical insights for preventing and controlling bacterial diseases in monkeys.

Acinetobacter species possess typical Gram-negative cell walls; however, due to challenges associated with decolorization that allow crystal violet retention, misidentification as Gram-positive cocci may occur [11]. Additionally, distinguishing between different Acinetobacter species morphologically is often difficult [23]. Consequently, 16S rRNA gene detection serves as a rapid and reliable method for identifying Acinetobacter species or strains [24]. Sequence analysis revealed that the strain isolated in this study exhibited high similarity to known *A. lwoffii* strains, and the homology reached up to 99.8%. However, the 16S rRNA gene, as a functional component of ribosomal, is subject to strong purifying selection. The sequence divergence among closely related species within the genus Acinetobacter is often less than 1%, which falls significantly below the conventional threshold of “98.5% similarity = same species” [25]. For instance, the 16S sequences of *A. lwoffii* and members of the *Acinetobacter calcoaceticus-Acinetobacter baumannii* (ACB) complex exhibit considerable overlap and cannot be differentiated based solely on this gene [26]. Furthermore, the genome of Acinetobacter contains multiple copies of 16S rRNA; although these genomes are overall similar, they display micro-genomic heterogeneity. Gene conversion may further homogenize differences between these copies, obscuring signals indicative of species differentiation and increasing identification ambiguity [27]. Additionally, while 16S rRNA reflects the evolutionary history of a single gene, closely related Acinetobacter species may experience incomplete lineage sorting (ILS) due to recent rapid radiative divergence; thus, a single-gene tree fails to accurately represent true species relationships. More comprehensive markers, such as the core genome, are necessary for precise classification [28]. Therefore, employing this method to distinguish closely related species within the genus Acinetobacter presents certain limitations.

Human infections caused by Acinetobacter frequently arise among patients suffering from chronic conditions such as diabetes mellitus, chronic obstructive pulmonary disease (COPD), renal disease, heavy smoking habits, or excessive alcohol consumption [29]; moreover, certain cases of peritonitis have been linked to Acinetobacter infections resulting from peritoneal dialysis procedures [30]. However, Takos and Thomas [2] reported that *Escherichia coli* and *Clostridium perfringens* also contribute to peritonitis in monkeys. Although *A. lwoffii* has a relatively weak pathogenic ability, it poses a significant threat to animals and humans with weakened immune systems [31]. Pathological analysis indicated that the cynomolgus monkey in this case suffered from peritonitis. At the same time, through the investigation of the animal’s past medical history and experimental data, it was shown that the animal had undergone multiple egg retrievals and embryo transfer surgeries. Therefore, researchers speculated that when the immunity of this cynomolgus monkey declined, the introduction of pathogenic bacteria during multiple experimental operations was an important reason for the animal’s infection with *A. lwoffii* and the occurrence of peritonitis.

In human cases involving Acinetobacter-related peritonitis, treatment typically involves appropriate antibiotics; however, the sensitivity of Acinetobacter to antibiotics has become an important concern [32]. Cao et al. [14] reported that Acinetobacter demonstrates resistance to cephalosporins, aminoglycosides and β-lactam drugs while remaining sensitive only towards select fluoroquinolones and tetracyclines. The isolated strains in this study did not exhibit resistance to all the antibiotics involved; the possible reasons, besides the different types of antibiotics selected for the study, may also be related to the type of Acinetobacter. Darby et al. [33] reported that compared with *Acinetobacter baumannii* (*A. baumannii*), *A. lwoffii* has a reduced ability to develop antibiotic resistance. This might be because *A. lwoffii* encodes only one tripartite RND system. RND efflux pumps play a significant role in the development of drug resistance. For instance, in other types of Gram-negative bacteria, the removal of efflux pumps reduces the frequency of mutation selection [34]. Harfe and Jinks-Robertson [35] speculated that another potential mechanism for the lack of resistance in *A. lwoffii* might be the more rigorous DNA repair mechanism in *A. lwoffii*. However, whether the current lack of drug resistance of *A. lwoffii* in clinical practice is due to the absence of an excretion system and/or more rigorous DNA repair and defense, or other factors, requires more research to clarify. Although the extensive antibiotic sensitivity of *A. lwoffii* enables it to achieve successful clinical outcomes, there are also sporadic cases of drug resistance, highlighting the possibility of drug resistance [36]. Rapid disease progression and weak immunity may be the primary factors contributing to mortality in the animal in this study.

The pathogenic mechanism of Acinetobacter on non-human primates remains unclear. In this study, researchers reported the first case of *A. lwoffii* infection in a cynomolgus monkey associated with peritonitis. This research provides technical support for pathogen screening and health monitoring of *A. lwoffii* in laboratory monkeys. However, it is important to acknowledge certain limitations of this study, including the restricted number of isolated strains, which do not adequately represent the prevalence characteristics and antibiotic resistance profiles of strains found in monkey populations across different regions or breeding conditions. Furthermore, investigations into the pathogenic mechanisms and resistance mechanisms associated with these strains require further research.

## 5. Conclusions

In conclusion, to clarify the cause of death of a cynomolgus monkey, a strain of bacteria, MF080196, was isolated from the abdominal cavity of the cynomolgus monkey with peritonitis in this study. Through bacterial isolation and culture, morphological observation, biochemical tests and molecular biological analysis, it was identified as *A. lwoffii*. The isolated strain demonstrated lethality when tested using an intraperitoneal mouse challenge model. Notably, this strain exhibited sensitivity to 19 drug-sensitive tablets evaluated during the study. These findings provide valuable insights for clinical diagnosis and treatment strategies concerning *A. lwoffii* infections in non-human primates.

## Figures and Tables

**Table 1:** Biochemical reaction results of isolated bacteria.

Biochemical Project	Results	Biochemical Project	Results
Mannitol	−	Urea	−
Hydrogen sulfide (H_2_S)	−	Glucose gas production	−
Phenylalanine	−	Lysine	−
Gluconate	−	Ornithine	−
Indigo substrate	−	Raffinose	−
VP (Voges–Proskauer test)	−	Sorbitol	−
MR (Methyl-Red test)	−	Scutellarin	−
Citrate	−	Xylose	−
Catalase	+	Oxidase	−

“+”indicated positive; “−“indicated negative.

**Table 2:** Results of drug sensitivity testing of *A. lwoffii* strains isolated from cynomolgus monkey.

Antibiotics	Medication Dose: µg/Tablet	IZD/mm (Single Test)	Sensitivity	Judgment Standard of Inhibition Zone Diameter (mm)		
				Resistant	Medium Sensitivity	Highly Sensitive
Ceftriaxone	30	26	S	≤13	14–22	≥23
Amikacin	30	26	S	≤14	15–16	≥17
Gentamicin	10 ± 2.5	27	S	≤12	13–14	≥15
Ampicillin	10	26	S	≤13	14–16	≥17
Cefazolin	30	16	I	≤14	15–17	≥18
Ceftazidime	30	24	S	≤17	18–20	≥21
Levofloxacin	5	27	S	≤13	14–16	≥17
Cotrimoxazole	23.75	27	S	≤10	11–15	≥16
Ciprofloxacin	5	31	S	≤15	16–20	≥21
Norfloxacin	10	26	S	≤12	13–16	≥17
Erythromycin	15	26	S	≤13	14–22	≥23
Rifampicin	5	25	S	≤6	7–9	≥10
Amoxicillin	10	28	S	≤5	6–9	≥10
Furazolidone	300	12	S	≤5	6–9	≥10
Cefoperazone	75	38	S	≤15	16–20	≥21
Cefadroxil	30	28	S	≤13	14–16	≥17
Ofloxacin	5	27	S	≤12	13–15	≥16
Imipenem	10	39	S	≤19	20–22	≥23
Meropenem	10	31	S	≤19	20–22	≥23
Azithromycin	15	34	S	≤12	–	≥13

S: susceptible, I: intermediate.

## Data Availability

The original contributions presented in this study are included in the article. Further inquiries can be directed to the corresponding author(s).

## Supplementary Materials

https://www.mdpi.com/article/10.3390/vetsci13020170/s1, Figure S1: Original PCR image.
