# Supplementary figures and images for: Molecular Identification and Drug Sensitivity Test of Acinetobacter lwoffii from Cynomolgus Monkey with Peritonitis
Source: Vet Sci. 2026 Feb 9;13(2):170. doi: 10.3390/vetsci13020170 (PMC12944971; doi:10.3390/vetsci13020170)

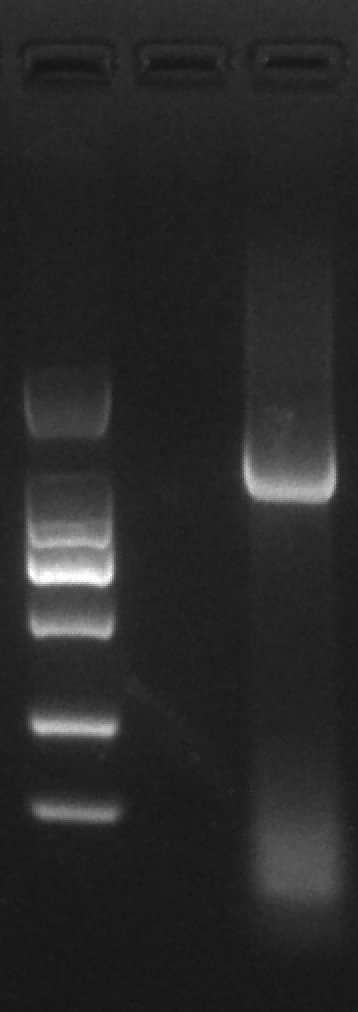

Supplement: Supplementary file 1 [file vetsci-13-00170-s001.zip › Figure S1-Original PCR image.jpg]
